# *Hovenia dulcis* Thunb Extract and Its Ingredient Methyl Vanillate Activate Wnt/β-Catenin Pathway and Increase Bone Mass in Growing or Ovariectomized Mice

**DOI:** 10.1371/journal.pone.0085546

**Published:** 2014-01-22

**Authors:** Pu-Hyeon Cha, Wookjin Shin, Muhammad Zahoor, Hyun-Yi Kim, Do Sik Min, Kang-Yell Choi

**Affiliations:** 1 Translational Research Center for Protein Function Control, College of Life Science and Biotechnology, Yonsei University, Seoul, Korea; 2 Department of Biotechnology, College of Life Science and Biotechnology, Yonsei University, Seoul, Korea; 3 Department of Molecular Biology, College of Natural Science, Pusan National University, Pusan, Korea; Oklahoma State University, United States of America

## Abstract

The Wnt/β-catenin pathway is a potential target for development of anabolic agents to treat osteoporosis because of its role in osteoblast differentiation and bone formation. However, there is no clinically available anti-osteoporosis drug that targets this Wnt/β-catenin pathway. In this study, we screened a library of aqueous extracts of 350 plants and identified *Hovenia dulcis* Thunb (HDT) extract as a Wnt/β-catenin pathway activator. HDT extract induced osteogenic differentiation of calvarial osteoblasts without cytotoxicity. In addition, HDT extract increased femoral bone mass without inducing significant weight changes in normal mice. In addition, thickness and area of femoral cortical bone were also significantly increased by the HDT extract. Methyl vanillate (MV), one of the ingredients in HDT, also activated the Wnt/β-catenin pathway and induced osteoblast differentiation *in vitro*. MV rescued trabecular or cortical femoral bone loss in the ovariectomized mice without inducing any significant weight changes or abnormality in liver tissue when administrated orally. Thus, natural HDT extract and its ingredient MV are potential anabolic agents for treating osteoporosis.

## Introduction

Bone homeostasis is maintained by a balance between osteoblast-mediated bone formation and osteoclast-mediated bone resorption [1]. An imbalance in bone homeostasis causes various diseases including osteoporosis, which is characterized by low bone mass and increased risk of bone fractures [2]. Osteoporosis frequently occurs in the elderly and in menopausal woman, and the most wildly used anti-osteoporosis drugs are anti-resorptive bisphosphonates that inhibit the activity of osteoclasts. Bisphosphonates, including alendronate (ALN), efficiently prevent bone loss; however, they have various adverse effects including upset stomach, inflammation of the esophagus, and osteonecrosis of the jaw [3]–[5]. In addition, bisphosphonates also elevate the risk of bone fractures caused by accumulation of microfractures [6]. Another class of anti-resorptive drugs includes estrogens and selective estrogen receptor modulators (SERMs). However, sustained treatment with these therapies results in higher risk of breast cancer, uterine bleeding, and cardiovascular events [7]. These adverse effects of anti-resorptive drugs warrant development of anabolic agents to treat osteoporosis.

Unlike anti-resorptive agents, anabolic agents stimulate proliferation or differentiation of osteoblasts, and consequently, improve both quality and quantity of bone [8]. Parathyroid hormone (PTH) drugs such as PTH (1–34) and PTH (1–84) are only approved anabolic agents by the US Food and Drug Administration (FDA) and/or European Union (EU) [9]. PTH drugs increase BMD and decrease vertebral fracture risk in humans compared to the anti-resorptive drugs, ALN and a SERM raloxifene, respectively [10], [11]. However, these protein drugs can be applied by only subcutaneous injection on the daily basis, and relatively expensive. In addition, treatment with PTH drugs is approved for a maximum of 2 years for continuous usage in humans, because of increased osteosarcoma incidence and hyperparathyroidism [8], [12]. Therefore, novel and safe anabolic agents for treating osteoporosis are increasingly needed.

The Wnt/β-catenin signaling pathway is of interest as a novel therapeutic target for development of osteoporosis treatment. Growing evidence suggests that activation of this pathway increases osteoblast differentiation and subsequent bone formation, while suppressing osteoclastogenesis [13], [14]. The drugs activating Wnt/β-catenin pathway are often suspected to induce human cancers, because aberrant activation of this pathway is known to relate various cancer developments. However, involvement of the Wnt/β-catenin pathway activation in osteosarcoma occurrence was not reported unlike bone morphogenetic protein (BMP) or transforming growth factor signaling [15]–[17]. Wnt/β-catenin pathway is inactive in osteosarcoma, and inhibition of this pathway contributes to the tumorigenesis of osteosarcoma [18]. Therefore, activation of the Wnt/β-catenin pathway is relatively safe for bone anabolic agent development compared with PTH or BMP pathway activators. Inhibitors for GSK3β or antibodies against dickkopf-1 (DKK1), sclerostin and secreted frizzled-related protein-1 (sFRP1), antagonists of Wnt/β-catenin pathway, increase bone formation and bone mass in mice, rats or humans [19]. A sclerostin neutralizing antibody entered phase I clinical trials in 2007 as the first osteoporosis treatment candidate in the Wnt/β-catenin pathway. However, clinically available osteoporosis drugs, especially small molecular drugs that target Wnt/β-catenin pathway are not available.

Natural products such as plant extracts and their individual ingredients have been used traditionally to treat various diseases including obesity and inflammation in eastern Asia and western Africa [20], [21]. These natural products are regarded as relatively safe for drug development and are increasingly being used in medicines. The global market of medicinal plants was estimated at approximately 83 billion US dollars in 2008, and the World Health Organization reports that over 80% of the world's population use natural products for medicinal treatment of primary health care needs [22], [23]. This resource, however, has not been fully exploited and many more natural products remain to be identified.

In this study, we searched plant extracts that activate Wnt/β-catenin signaling pathway and induce osteoblast differentiation. Through the screening of plant extracts library, we identified an extract from *Hovenia Dulcis* Thunb (HDT) as an activator of the Wnt/β-catenin pathway, and characterized its ability to modulate osteoblast differentiation and bone mass *in vitro* and *in vivo*, respectively. We determined that methyl vanillate (MV), an ingredient of HDT, activates the Wnt/β-catenin pathway and is involved in osteoblast differentiation. The non-toxic concentration of MV rescued femoral bone loss in ovariectomized mice after oral administration, and the effect was equivalent to that of intraperitoneal PTH (1–34) injection. HDT extract and the small molecule MV have potential for development as anabolic agents to treat osteoporosis.

## Materials and Methods

### Cell culture, transfection and reagents

Human embryonic kidney 293 (HEK293) cells and HEK293 reporter cells (containing the chromosomally incorporated TCF reporter (TOPflash) gene) [24] were grown in Dulbecco's Modified Eagle Medium (DMEM; Gibco BRL, Carlsbad, CA, USA) supplemented with 10% fetal bovine serum (FBS; Gibco BRL), and 100 U/ml penicillin G and 100 µg/ml streptomycin (Gibco BRL). Calvarial osteoblasts were extracted from calvaria of ICR mice at postnatal day 4. The calvaria were digested with 0.32 mg/ml collagenase type II (Worthington, Lakewood, NJ, USA) for 20 min at 37°C, and the extracted cells were collected by centrifugation at 1500 RPM for 2 min. These steps were repeated five times. This calvarial cells and mouse osteoblastic cell line, MC3T3E1, were cultured in basic medium (α-Minimum Essential Medium (α–MEM; Gibco BRL) supplemented with 10% FBS and antibiotics). For differentiation of osteoblast cells, 100 µg/ml ascorbic acid (Sigma Aldrich, St. Louis, MO, USA) and 10 mM β-glycerophosphate (Sigma Aldrich) were added to the basic media. Transfection of plasmid or siRNA (β-catenin and control GFP; Bioneer, Daejeon, Korea) was performed with Lipofectamine Plus (Invitrogen, Carlsbad, CA, USA) according to the manufacturer's instructions. Plant extracts, including the HDT extract and its 8 ingredients (vanillic acid (VA), methyl vanillate (MV), ferulic acid (FA), myricetin, taxifolin, 2,3,4-trihydrobenzoic acid (2,3,4-TA), dihydrokaempferol (DH) and gallocatechin (GC)) were purchased from the Korea Plant Extract Bank and Sigma Aldrich, respectively, and those were dissolved in dimethyl sulfoxide (DMSO; Sigma Aldrich) for *in vitro* studies.

### Reporter assay

For plant extracts screening, HEK293 reporter cells were seeded onto 96-well black polystyrene plates (Greiner Bio-One, Stonehouse, UK) at 2×10^4^ cells per well and grown for 24 h. Each plant extract or DMSO (control) was added at a final concentration of 1 µg/ml. After 24 h, firefly luciferase activity was measured and the relative reporter activity was determined by normalizing to the control [24]. For quantitative analysis of TOPflash activity induced by the HDT extract or its individual ingredients, HEK293 reporter cells were seeded into 24-well plates at a density of 5×10^4^ cells/well. After 24 h, the HDT extract (5 or 50 µg/ml) or the 8 ingredients (each 20 µM) were individually added to the cells for 24 h. Reporter activity was measured as described previously [24]. TOPflash and FOPflash assays were performed in calvarial osteoblasts. The pTOPflash or pFOPflash [25] vector was transfected with pCMV–β-galactosidase (β-gal; Clontech, Mountain View, CA, USA) reporter plasmids using Lipofectamine Plus. After 24 h, HDT extract (5 µg/ml) or DMSO (control) was added to the calvarial osteoblasts for another 24 h. Luciferase activity was measured in whole cell lysates and normalized to the internal control β-gal.

### *Ex vivo* culture and morphometric analysis of mouse calvaria

Calvaria were extracted from ICR mice at postnatal day 4, and cultured on a grid in a 12-well plate with α–MEM for 24 h. Plant extracts or MV in differentiation media were treated to calvaria for 7 days, and those were changed with identical fresh media every 2 days. The calvaria were fixed in 4% paraformaldehyde (PFA) for 2 days, and decalcified in 4% HCl and 4% formic acid for 2 days. After decalcification, the calvaria were dehydrated, embedded in paraffin, and sagittally sectioned to a thickness of 4 µm (Leica Microsystems, Wetzlar, Germany) from the midline [26]. The sectioned tissues were rehydrated and hematoxylin and eosin (H&E) staining was performed.

### Animals and experimental treatments

ICR mice were purchased from KOATECH (Pyeongtaek, Gyeonggido, Korea), and animal care and experiments were carried out according to the guidelines of the Korean Food and Drug Administration. Protocols were reviewed and approved by the Institutional Review Board of Severance Hospital, Yonsei University College of Medicine (09-013). Animals were maintained under a 12 h light/12 h darkness cycle at 22–25°C in a conventional conditions and fed with standard rodent chow and water.

Normal 8-week-old male mice were given intraperitoneal (i.p.) injection of 200 mg/kg of HDT extract for 5 sequential days each week for 4 weeks. Each group included 5 mice, and the weight of mice was measured every 4 days. Calcein (10 mg/kg; Sigma Aldrich) was i.p. injected into at least 3 mice per a group at 15 days and 5 days prior to sacrifice, respectively. At 8 weeks of age, females were ovariectomized (OVX) under anesthesia with avertin (2,2,2-tribromoethanol; Worthington). Dorsal incisions, approximately 1-cm long, were made with dissection scissors into the dermal layer above both sides of the ovaries. The connection between the fallopian tube and the uterine horn was cut and the ovary was removed. The incision was then sutured with 3 single catgut stitches. Sham-operated (Sham) mice were used as controls. After 2 or 4 weeks, the OVX group was divided into several groups according to experimental designs (n = 5–7 per group). PTH (1–34) (80 µg/kg; BACHEM, Bubendorf, Switzerland) was i.p. injected into mice, while MV was dissolved in corn oil and orally applied. Treatment of both PTH (1–34) and MV was given for 5 sequential days each week for 4 weeks. Animals were sacrificed and organs were obtained for analysis.

### Bone histomorphometric analysis and Immunohistochemistry (IHC)

The tissues were fixed in 4% PFA for 3 days at 4°C for histological evaluation. After fixation, femurs were decalcified in 10% ethylenediaminetetraacetic acid (EDTA; Sigma Aldrich), dehydrated, embedded in paraffin and sectioned to 4 µm thickness (Leica Microsystem). The tissues were rehydrated and used for further analyses including H&E and IHC staining. Florescence IHC analyses were described previously [27]. For fluorescent IHC, sections were incubated with primary antibody (anti-β-catenin; Santa Cruz Biotechnology, Santa Cruz, CA, USA) overnight at 4°C, followed by incubation with anti-mouse Alexa Flour 488 (Life Technologies, Carlsbad, CA, USA; 1∶500) or anti-rabbit Alex Flour 555 (Life Technologies; 1∶500) secondary antibodies for 1 h at room temperature. The sections were then counterstained with 4′, 6-diamidino-2-phenylindole (DAPI; Sigma Aldrich, St. Louis, MO, USA) and mounted in Gel/Mount media (Biomeda Corporation, Foster City, CA, USA). All incubations were conducted in dark, humid chambers. The fluorescence signals were visualized using confocal microscopy (LSM510; Carl Zeiss Inc., Thornwood, NY) at excitation wavelengths of 488 nm (Alexa Fluor 488), 543 nm (Alexa Fluor 555) and 405 nm (DAPI). At least 3 fields per section were analyzed.

### Microcomputated tomography analysis and cortical bone measurement

After sacrifice, mouse femurs were harvested and stored in 70% ethanol (Duksan Pure Chemical Co., Ansan, Gyeonggido, Korea) until analysis. To analyze the femoral trabecular bone, standardized cone-beam microcomputed tomography (μCT) scanning of the right limb was performed using a μCT system for small animal imaging (Skyscan 1076; Skyscan, Kontich, Belgium). Scanning was performed with a 10 µm thick at the region of the distal femur from growth plate and extended proximally along the femur diaphysis. One hundred continuous slices were scanned and analyzed stating at 0.1 mm from the most proximal aspect of the growth plate until both condyles were no longer visible were selected for analysis. All trabecular bone from each selected slice was segmented for three dimensional reconstruction to calculate the bone parameters such as bone volume/tissue volume (BV/TV), trabecular number (Tb.N), trabecular separation (Tb.Sp), and trabecular thickness (Tb.Th) [28]. Cortical bone parameters such as outer diameter of x-axis (C.Od), outer diameter of y-axis, inner diameter or thickness (C.Th) were evaluated using NIS elements AR 3.1 software (Nikon) in μCT 3D images. The cortical bone area (C.Ar) was calculated by mathematical formula for ellipse by multiplication of x-axis diameter, y-axis diameter and pi (π) value.

### RNA extraction, cDNA synthesis and reverse transcriptase polymerase chain reaction (RT-PCR)

Total RNA was isolated using Trizol (Invitrogen) and 2 µg of total RNA was reverse transcribed using 200 U of reverse transcriptase (Invitrogen) according to the manufacturer's instructions. The following primer sets were used: *runt-related transcription factor 2* (*RUNX2*), forward 5′-GAGGCCGCCGCACGACAACCG-3′ and reverse 5′– CTCCGGCCCACAAATCTCAGA-3′; *bone morphogenetic protein 2* (*BMP2*), forward 5′-AGAGATGAGTGGGAAAACGG-3′ and reverse 5′-GAAGTCCACATACAAAGGGT-3′; *alkaline phosphatase* (*ALP*), forward 5′-GGGACTGGTACTCGGATAACGA-3′ and reverse 5′- CTGATATGCGATGTCCTTGCA-3′; *osteocalcin (OCN*), forward 5′-GCAGCTTGGTGCACACCTAG-3′ and reverse 5′-ACCTTATTGCCCTCCTGCTT-3′; *receptor activator of nuclear factor kappa-B ligand* (*RANKL*), forward 5′-CCAGTGAAGCAGCAGCCAGC-3′ and reverse 5′-CCCTCTCATCAGCCCTGTCC-3′; *osteoprotegerin* (*OPG*), forward 5′-ACGGACAGCTGGCACACCAG-3′ and reverse 5′-CTCACACACTCGGTTGTGGG-3′; *GAPDH*, forward 5′-CCATGGAGAAGGCTGGGG′ and reverse 5′–CAAAGTTGTCATGGATGACC-3′.

### Immunoblotting

Cells were washed with ice-cold phosphate-buffered saline (PBS; Gibco BRL, Carlsbad, CA, USA) and lysed in radio immunoprecipitation assay (RIPA) buffer (Millipore, Bedford, MA, USA). Proteins were separated on a 6–15% sodium dodecyl sulfate (SDS) polyacrylamide gel and transferred to a nitrocellulose membrane (Whatman, Florham Park, NJ, USA). Immunoblotting was performed with the following primary antibodies: anti-β-catenin (Santa Cruz Biotechnology, Santa Cruz, CA, USA) and anti-α-tubulin (Oncogene Research Products, Cambridge, MA, USA). Horseradish peroxidase-conjugated anti-mouse (Cell Signaling, Beverly, MA, USA) or anti-rabbit (Bio-Rad Laboratories, Hercules, CA, USA) antibodies were used as secondary antibodies.

### Immunofluorescence staining

Calvarial osteoblasts were seeded onto a coverslip in 12-well plates at a density of 3×10^4^ cells/well. After 24 h, HDT extract or MV was added for another 24 h and the cells were subsequently fixed with 4% PFA for 10 min. Cells were permeabilized with 0.2% Triton X-100 for 15 min and incubated for 30 min with 5% bovine serum albumin (BSA) blocking solution. The cells were incubated with the primary antibody (β-catenin) overnight at 4°C and then washed with PBS three times. Cells were then incubated with Alexa Fluor 502-conjugated IgG secondary antibody (Life Technologies, Carlsbad, CA, USA) was incubated for 1 h, followed by incubation with 4′, 6-diamidino-2-phenylindole (DAPI; Sigma Aldrich, St. Louis, MO, USA) for 5 min. The cells were mounted in Gel/Mount media (Biomeda Corporation, Foster City, CA, USA). The fluorescence signal was captured using confocal microscopy (LSM510; Carl Zeiss Inc., Thornwood, NY). To measure the fluorescence signal, we detected intensities of β-catenin in the fluorescence staining images using NIS elements AR 3.1 software (Nikon).

### Cytotoxicity assay

Calvarial osteoblasts were plated at a density of 5×10^3^ cells per 24-well plate. The cells were then treated with DMSO (control) or HDT extract for 72 h. Next, 3-(4,5-Dimethylthiazol-2-yl)-2, 5-diphenyltetrazolium bromide (MTT; AMRESCO, Solon, Ohio, U) was added to each well at a concentration of 0.25 mg/ml. After incubation for 2 h at 37°C, insoluble purple formazan was obtained by media removal. The formazan was dissolved in 1 ml DMSO for 1 h. The absorbance of the formazan product was measured at 590 nm.

### Alkaline phosphatase (ALP) assay and staining

Calvarial osteoblasts were seeded in 24-well plates at a density of 5×10^4^ cells/well. The HDT extract or MV in differentiation media was added to the cells for 5 days. The basic media was used as a negative control. The media with DMSO alone, HDT extract, or MV was changed after 3 days. To measure ALP activity, the cells were washed with cold PBS and lysed in 55 µl of buffer containing 0.2 M Tris-HCl (pH 8.0) and 0.1% Triton X-100. After centrifugation at 13,000 RPM for 15 min at 4°C, the supernatant (15 µl) was incubated with 30 µl of *p*-nitrophenylphosphate (pNPP, Sigma Aldrich), a substrate used to quantify ALP activity. To stop the reaction, 20 µl of 0.2 N NaOH was used and the absorbance was read at 405 nm. Whole cell lysates were analyzed for protein concentration using the Bradford assay (Bio-Rad Laboratories), and ALP activity was normalized to the protein concentration. ALP was stained using the TRACP & ALP double-stain kit (Takara Bio Inc., Shiga, Japan) according to the manufacturer's instructions.

### Alizarin Red S staining

Calvarial osteoblasts were seeded into 24-well plates at a density of 5×10^4^ cells/well. The HDT extract or MV in differentiation medium was treated into the cells for 21 days. Media changes occurred every 3 days. At the end of incubation period, the cells were washed with cold PBS and fixed with 4% PFA for 10 min. The cells were stained with 40 mM Alizarin staining solution (pH 4.2; Sigma Aldrich) for 30 min at room temperature and washed with water three times. Colorimetric staining was extracted and quantified at 550 nm [29].

### Statistical analyses

Data are presented as mean ± standard deviation (S.D.) unless otherwise indicated. Significance was analyzed using a Student's *t* test.

## Results

### Identification of HDT extract as an activator of Wnt/β-catenin signaling

**Figure 1:**
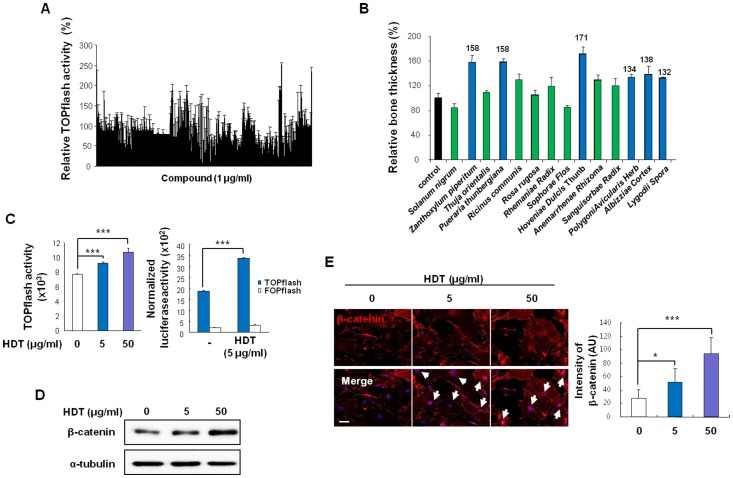
Identification of *Hovenia dulcis* Thunb (HDT) extract as an activator of Wnt/β-catenin signaling pathway. (A) Each of the 350 plant extracts (1 µg/ml each) was added to HEK293 reporter cells for 24 h, and TOPflash activity was measured. (n = 3). (B) Fourteen plant extracts, which showed increased TOPflash activity compared with control, were subjected to calvaria *ex vivo* assay. Of 14 plant extracts, six plant extracts, which increased the thickness of the *ex-vivo* cultured calvaria, were marked by blue bars (n = 2). (C–E) HDT extract was added to HEK293 reporter cells (C) or calvarial osteoblasts (D, and E) for 24 h. (C) Luciferase activity of HEK293 reporter cells (left) and calvarial osteoblasts transfected with TOPflash or FOPflash (right) was measured, respectively (n = 3). (D–E) β-catenin proteins were detected by immunoblotting (D) and immunofluorescence staining (E, left), respectively (white arrows indicate nuclear localized β-catenin). Scale bars, 50 µm. Intensities of β-catenin were measured from the immunofluorescence staining images (E, right) (n>3). (C, and E) *p<0.05, ***p<0.001 *versus* control.

To identify plant extracts that activate the Wnt/β-catenin pathway, we screened aqueous extracts of 350 plants using the HEK293 reporter cells containing TOPflash. Fourteen plant extracts showed the >150% TOPflash activity compared with the control (Figure 1A). Using *ex vivo* calvaria assays, we found that six plant extracts stimulated bone formation (>130% compared with the control). The HDT extract revealed the highest increase in bone thickness of calvaria among the 6 plant extracts (Figure 1B). We further confirmed that the HDT extract dose-dependently increased TOPflash activity in HEK293 reporter cells and increased the activity of TOPflash but not the FOPflash harboring mutant TCF binding site in calvarial osteoblasts (Figure 1C). Expression of β-catenin and translocation of β-catenin into nuclei were also dose-dependently increased with exposure to the HDT extract in calvarial osteoblasts (Figure 1D, and E). HDT extract as high as 50 µg/ml was not cytotoxic in calvarial osteoblasts for 72 h (Figure S1). HDT extract activates the Wnt/β-catenin pathway without significant toxicity in calvarial osteoblasts.

### HDT extract induces osteoblast differentiation of primary calvarial osteoblasts

**Figure 2:**
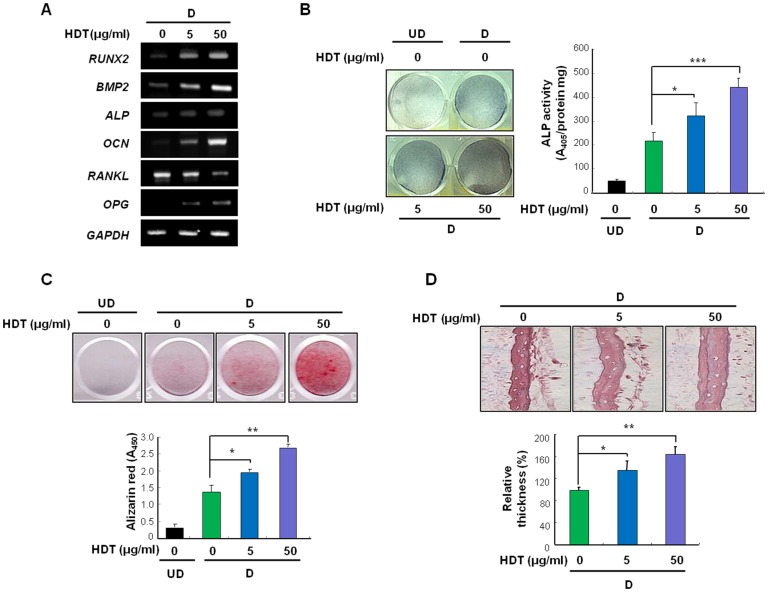
HDT extract has osteogenic effects on calvarial osteoblasts and mouse calvaria. (A–C) Calvarial osteoblast cells were treated with HDT extract or DMSO (control) in osteogenic differentiation (D) or the basic undifferentiation (UD) medium. (A) Calvarial osteoblasts were treated with HDT extract for 3 days followed by harvesting for RT-PCR analyses. (B) Calvarial osteoblasts were treated with HDT extract for 5 days and then subjected to ALP staining (left) or ALP activity measurements (right). (C) Calvarial osteoblasts were treated with HDT extract for 21 days. The cells were then stained with Alizarin Red S solution (up) and quantification was performed by measuring absorbance at 450 nm (n = 3; down). (D) Calvaria were treated with HDT extract in osteogenic differentiation (D) media for 7 days. Thickness of the calvaria was assessed by H&E staining (up) and quantified (down; n = 3). (B–D) *p<0.05, **p<0.01, ***p<0.001 *versus* control of differentiation medium.

Because activation of the Wnt/β-catenin pathway stimulates osteoblast differentiation, we investigated whether HDT extract can induce osteoblast differentiation of primary calvarial osteoblasts. HDT extract dose-dependently increased mRNA levels of the osteoblast differentiation markers such as *RUNX2, BMP2, ALP* and *OCN* (Figure 2A). Gene expression of two genes related to osteoclast activity, *RANKL*, a secreted osteoclastogenesis activator, and *OPG*, a secreted osteoclastogenesis inhibitor, was reduced and increased dose-dependently, respectively (Figure 2A). HDT extract also elevated ALP activity compared with the control in a dose-dependent manner (Figure 2B). Alizarin Red S staining revealed that the HDT extract induced terminal osteogenic differentiation (Figure 2C). We confirmed the effect of HDT extract on bone formation *ex vivo* using calvaria from neonatal mice. H&E staining showed that HDT extract increased calvaria thickness in a dose-dependent manner (Figure 2D).

### Intraperitoneal application of HDT extract stimulated bone mass of normal ICR mice

**Figure 3:**
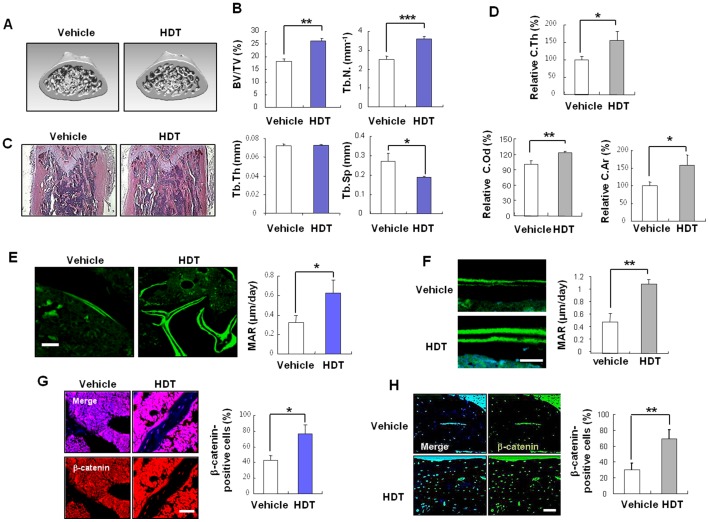
HDT extract enhances bone mass in normal mice. (A–H) HDT extract (200 mg/kg) was i.p. injected into 8-weeks-old male mice (n = 5) and the femurs were analyzed. (A) The representative μCT images are shown. (B) Trabecular bone parameters, such as BV/TV (%), Tb.N. (mm^−1^), Tb. Th (mm) and Tb.Sp (mm) from the μCT analysis are presented. (C) Photomicrographs of H&E stained-femur from vehicle- and HDT extract-treated mice are shown (original magnification: ×40). (D) Cortical bone parameters such as C.Th, C.Od, and C.Ar were measured from μCT 3D images and were normalized by the values of vehicle, respectively. (E–F) Calcein double staining (left) and mineral appositional rate (MAR; right) of trabecular bone (E) and endocortical surface (F) at femurs (n = 3). Scale bars, 20 µm. (G–H) Florescence staining of β-catenin (left) in the femoral trabecular (G) and cortical bones (H) and quantification data are shown (n = 3; right). Scale bars, 50 µm. (B, and D–H) *p<0.05, **p<0.01, ***p<0.001 *versus* vehicle.

To investigate the effects of HDT extract on bone mass *in vivo*, 200 mg/kg of HDT extract was i.p. injected into 8-week-old male mice for 4 weeks. Femurs from mice injected with vehicle or HDT extract were analyzed using μCT generated 3D images (Figure 3A). HDT extract increased trabecular bone parameters such as BV/TV and Tb.N, but not Tb.Th (Figure 3B). Reversely, Tb.Sp was significantly reduced by HDT extract treatment (Figure 3B). Increase in trabecular bone by HDT extract treatment was also shown by H&E staining (Figure 3C).

Trabecular bone is related to metabolism and strength of bone. Cortical bone maintains the correct architecture and stiffness of bone [30]. Both trabecular and cortical bone volumes or thickness were increased in the mice harboring the Wnt/β-catenin pathway activation (GSK3β^+/−^, Sfrp3^−/−^, or Sost^−/−^ mice) [31]–[33]. To evaluate effects of HDT extract on femoral cortical bone, we analyzed cortical bone parameters such as outer diameter and thickness from the μCT 3D images. HDT extracts stimulated increment of thickness and area of femoral cortical bones as well as those of trabecular bones (Figure 3D). Calcein double labeling showed dynamic increase in bone formation of femoral trabecular (Figure 3E) or cortical bones (Figure 3F) of HDT extract-treated mice compared with those of the vehicle-treated mice. Compared with the vehicle-treated mice, we also confirmed that HDT extract-treated mice showed an increased expression of β-catenin in trabecular and cortical bones of femurs, respectively (Figure 3G, and H). Overall, HDT extract activated the Wnt/β-catenin pathway and increased femoral bone mass through elevation of bone formation in normal mouse model. Significant weight differences were not observed between the vehicle and HDT extract-treated groups (Figure S2).

### MV induced osteoblast differentiation of primary calvarial osteoblasts

**Figure 4:**
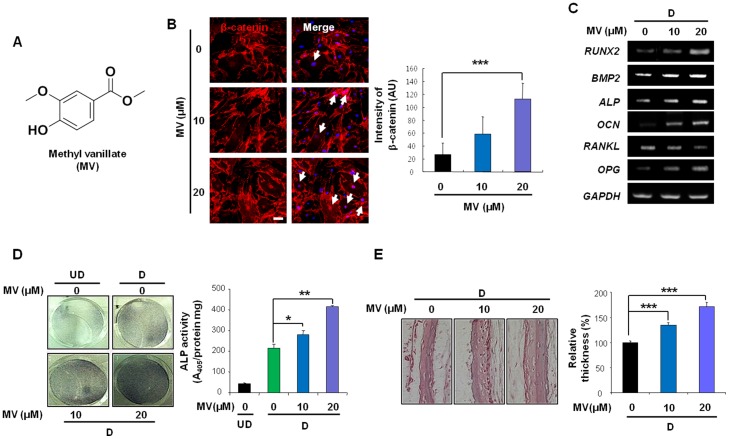
MV activates the Wnt/β-catenin signaling pathway and induces calvarial osteoblast differentiation. (A) A structure of MV. (B) Immunofluorescence staining of β-catenin in calvarial osteoblasts is shown (left, white arrows indicate nuclear β-catenin). Scale bars, 50 µm. Intensities of β-catenin were measured (right, n>3). (C–E) Osteogenic differentiation (D) or the basic (UD) medium was used. (C) Calvarial osteoblasts were treated with MV for 72 h and mRNA levels of the indicated genes were analyzed. (D) Calvarial osteoblasts treated with MV were stained for ALP (left) and ALP activity was measured (n = 3; right). (E) Calvaria isolated from postnatal day 4 mice were incubated with MV for 7 days. H&E staining revealed the thickness of the calvaria (left), and the thickness was quantified (n = 3; right). (B, D–E) *p<0.05, **p<0.01, ***p<0.001 *versus* control of differentiation medium.

To identify which HDT ingredients activate the Wnt/β-catenin pathway and induce osteoblast differentiation, we obtained 8 available ingredients that make up HDT; VA, MV, FA, myricetin, tasifolin, 2,3,4-TA, DH and GC [34]–[36]. Of these ingredients, MV, 2,3,4-TA and GC increased TOPflash activity in HEK293 reporter cells (Figure S3A) and MV most significantly increased the ALP activity (Figure S3B). MV did not cause any significant cytotoxicity when treated to calvarial osteoblasts as high as 20 µM for 72 h (Figure S4). Therefore, we chose MV for further characterization of its osteogenic capacity (Figure 4A). We observed that MV increased the expression and nuclear translocation of β-catenin in calvarial osteoblasts (Figure 4B). Like the HDT extract, MV increased the expression of differentiation markers *RUNX2, BMP2, ALP*, and *OCN* in a dose-dependent manner (Figure 4C). MV decreased and increased the expression of *RANKL* and *OPG*, respectively (Figure 4C). We confirmed that MV dose-dependently elevated ALP activity in calvarial osteoblasts (Figure 4D). Furthermore, calvarial thickness was dose-dependently increased with MV treatment (Figure 4E). Collectively, MV increased the β-catenin expression and induced osteoblasts differentiation, leading to the enhanced bone formation *ex vivo*. In addition, siRNA-mediated β-catenin knockdown abolished the increment of β-catenin (Figure S5A) and ALP activity (Figure S5B) by MV. These results indicated that the Wnt/β-catenin pathway signaling is involved in MV-induced ALP activation.

### Orally administrated MV rescues osteopenia-induced by ovariectomy

**Figure 5:**
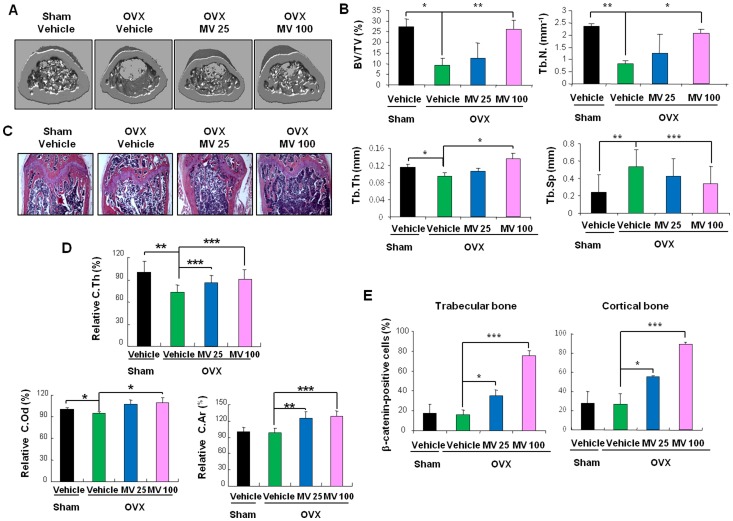
MV rescues bone loss induced by ovariectomy. (A–E) MV was orally administered to Sham- or OVX-mice for 4 weeks and femurs were used for further analysis (n = 5). (A) The representative μCT analysis images are presented. (B) The μCT analyses for femoral trabecular bone parameters are presented. (C) Femurs from the SHAM-vehicle, OVX-vehicle, or OVX-MV mice were stained with H&E (original magnification: ×40). (D) Cortical bone parameters were measured from μCT 3D images and were normalized by those of Sham-vehicle mice. (E) Florescence staining of β-catenin in the femoral trabecular and cortical bones (Figure S6) were quantified (n = 3). (B, D–E) *p<0.05, **p<0.01, ***p<0.001 between indicated samples.

To investigate whether MV reverses bone loss *in vivo*, we performed ovariectomies on mice and induced bone loss for 2 weeks. Lower bone mass in vehicle-treated OVX mice was confirmed through bone histomorphometric analyses by μCT (Figure 5A). Trabecular bone parameters such as BV/TV, Tb.N and Tb.Th were significantly reduced in OVX-vehicle mice compared with Sham-vehicle mice. Oral administration of MV rescued the reduced parameters in a dose-dependent manner (Figure 5B). In contrast, Tb.Sp was increased in OVX-vehicle mice. Following MV treatment, this parameter decreased in dose-dependent fashion (Figure 5B). We observed increase of trabecular bone volume at femurs with MV treatment by H&E staining (Figure 5C). MV also showed anabolic effects on femoral cortical bones (Figure 5D). MV dose-dependently elevated the expression of β-catenin in femoral trabecular and cortical bones, respectively (Figure 5E, Figure S6A, and B), however, no significant difference was observed in the expression of β-catenin between the vehicle-treated Sham and vehicle-treated OVX mice (Figure 5E).

The body weights of the MV-treated mice did not significantly differ compared to those of non-treated mice (Figure S7A). No discernible abnormalities were observed in H&E-stained liver tissue of MV-treated mice (Figure S7B). Because estrogen is limited in clinical applications due to its tumor-promoting characteristics, we checked whether MV has estrogenicity by assessing the wet weights and histomorphometry of the uterus [37]. The weight of wet uterine in OVX-mice was reduced by 84% compared with that of the Sham-mice, however, oral administration of MV did not exhibit increase in uterine weights compared to OVX-vehicle-treated mice (Figure S7C). We also confirmed that MV does not have any estrogenic effects by histomorphometric analyses showing maintenance of atrophic histological characteristics in the uterine of MV-treated OVX mice (Figure S7D). Overall, MV rescued bone loss induced by OVX without resulting in any significant changes of the weights or status of liver and uterine tissue.

### MV rescued bone loss in OVX-mice as efficiently as PTH drug

**Figure 6:**
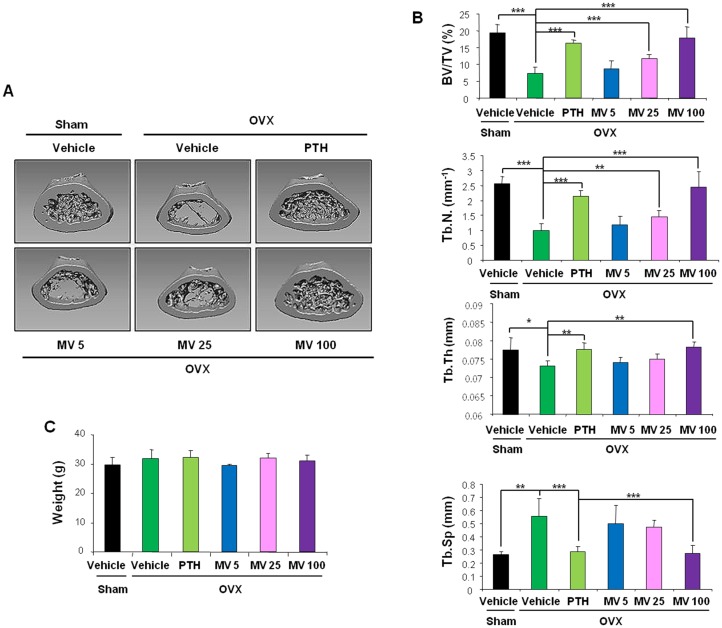
Effects of MV or PTH on the bone loss induced by ovariectomy. (A–D) MV or PTH (80 µg/kg) was administered to the Sham- or OVX-mice. The femurs were used for further analyses (n = 5–7). (A) The representative μCT analysis images are presented. (B) Femoral trabecular bone parameters were obtained for μCT analyses *p<0.05, **p<0.01, ***p<0.001 between indicated samples. (C) The relative weights of mice after final treatment with PTH or MV are shown.

We compared the efficacies of MV with PTH (1–34) in bone formation because PTH (1–34) is the only anabolic agent approved by the FDA for osteoporosis treatment [38]. We performed ovariectomies on mice and allowed them to develop severe osteopenia over a period of 4 weeks to induce (Figure 6A). In these mice, MV increased the BV/TV, Tb.N and Tb.Th, while decreasing Tb.Sp similar to PTH (1–34) treatment in OVX-mice compared to vehicle-treated OVX-mice (Figure 6A, and B). Interestingly, orally administered MV at 100 mg/kg revealed equivalent effects on bone mass to those observed with i.p. injection of PTH (1–34; 80 µg/kg) in femurs (Figure 6A, and B). No significant changes of weight were observed for mice used in the experiments (Figure 6C).

## Discussion

Because activation of the Wnt/β-catenin pathway induces osteoblast differentiation and bone mass, the pathway is of recent interest as a major target for development of anabolic agents to treat osteoporosis. Here, we screened 350 plant extracts to find novel activators of the Wnt/β-catenin pathway and identified HDT extract as a candidate for anabolic bone formation. HDT, also known as the Japanese raisin tree, has been used as a traditional medicine for treatment of liver diseases and for detoxification of alcoholic poisoning in eastern Asian countries such as Japan, China and Korea [39], [40]. The HDT extract also has anti-diabetic and anti-cancer effects, neuroprotective effects, and regenerative effects in damaged liver [36], [39]. However, the effect of HDT extract on skeletal physiology has not been reported. We investigated whether the HDT extract increases bone mass, following our identification of its function in the activation of Wnt/β-catenin pathway. We found that HDT extract induces osteogenic differentiation *in vitro* and increases femoral bone mass in *vivo*.

To identify the ingredients responsible for activating the Wnt/β-catenin pathway and inducing osteoblast differentiation, we tested the 8 known ingredients in HDT to determine whether they modulate Wnt/β-catenin signaling and osteoblast differentiation. Of those, we determined that MV is as the most effective active ingredient that increases osteoblast differentiation with activation of the Wnt/β-catenin pathway. MV is present in many plants, including the stem bark of *Zanthoxylum scandens* and HDT, and has been reported to have anti-platelet and anti-oxidant activity [36], [41]–[43]. Here, we showed that MV induces osteoblast differentiation *in vitro* and the oral administration of MV rescues femoral trabecular and cortical bone loss caused by OVX. Moreover, when orally administered at 100 mg/kg, MV possesses an efficacy similar to PTH (1–34; 80 µg/kg) [44] for recovering bone loss in OVX-mice. PTH drugs are a standard anabolic treatment for osteoporosis approved by the FDA. However, PTH drugs can only be administrated by injection and for only 2 years due to the risk of cancer [45]. Unlike PTH drugs, MV could be administrated orally and is considered safe because it is a natural plant-derived ingredient. We show that treatment with HDT extract or MV is not critically cytotoxic to calvarial osteoblasts and does not significantly affect the weights of mice used *in vivo* experiments. Medicinal plants and their active ingredients have been used in traditional medicines for centuries [20], [21], [46]. HDT extract from stem barks, leaves, and fruits is not cytotoxic to mammalian cells [36], [47]. HDT extract and its active ingredients are used as additives to food in Asian countries without inducing cytotoxicity [48], [49]. Thus, HDT extract and its active ingredient MV could be developed as potential anabolic agents for osteoporosis treatment.

## Supporting Information

Figure S1 **HDT extract did not affect cytotoxicity in calvarial osteoblasts.** Calvarial osteoblasts were treated with HDT extract for 72 h and the cytotoxicity was assessed by MTT assay (n = 3).
(TIF)

Figure S2 **HDT extract did not induce critical changes to the weights of the mice.** Weight difference of mice used in Figure 3 are presented (n = 5).
(TIF)

Figure S3 **MV is an ingredient in HDT responsible for Wnt/β-catenin pathway activation and increased osteoblasts differentiation.** (A) HEK293 reporter cells were treated with its 8 ingredients (20 µM) for 24 h, and subjected to TOPflash activity measurement (n = 3). (B) Calvarial osteoblasts were treated with MV, 2,3,4-TA, GC or FA for 3 days, and the cells were harvested for determination of ALP activity. ALP activity was normalized to the DMSO (control), and FA was used as a positive control (n = 3).
(TIF)

Figure S4 **MV did not affect cytotoxicity in calvarial osteoblasts.** Calvarial osteoblasts were incubated with MV for 72 h and the cytotoxicity of MV was determined by MTT assay (n = 3).
(TIF)

Figure S5 **MV increases ALP activity via activation of Wnt/β-catenin pathway in MC3T3E1 cell lines.** (A–B) The siRNA for β-catenin or a control (siRNA for GFP) was transfected into MC3T3E1 cells. After 12 h, MV was treated with differentiation media for 72 h and the cells were subjected to immunoblotting (A) and measurement of ALP activity (B; n = 3).
(TIF)

Figure S6 **MV increases β-catenin expression at femur.** (A–B) Data are shown from mice used in Fig. 5. (n = 5). Representative images of IHC staining for β-catenin in trabecular (A) and cortical (B) bone at femur. Scale bars, 50 µm.
(TIF)

Figure S7 **MV causes no critical abnormalities in weight, live, and uterine.** (A–D) Data are shown from mice used in Fig. 5. (n = 5). (A) The weights of mice were measured every 4 days. (B) Liver tissue from the mice was stained with H&E (CV; central veins). (C) The uteri were isolated from mice, and their wet weights were measured after sacrifice. (D) The uteri in (C) were subjected to H&E staining. (B, D) Scale bars, 100 µm.
(TIF)
