# Supplementary figures and images for: Hovenia dulcis Thunb Extract and Its Ingredient Methyl Vanillate Activate Wnt/β-Catenin Pathway and Increase Bone Mass in Growing or Ovariectomized Mice
Source: PLoS One. 2014 Jan 22;9(1):e85546. doi: 10.1371/journal.pone.0085546 (PMC3899039; doi:10.1371/journal.pone.0085546)

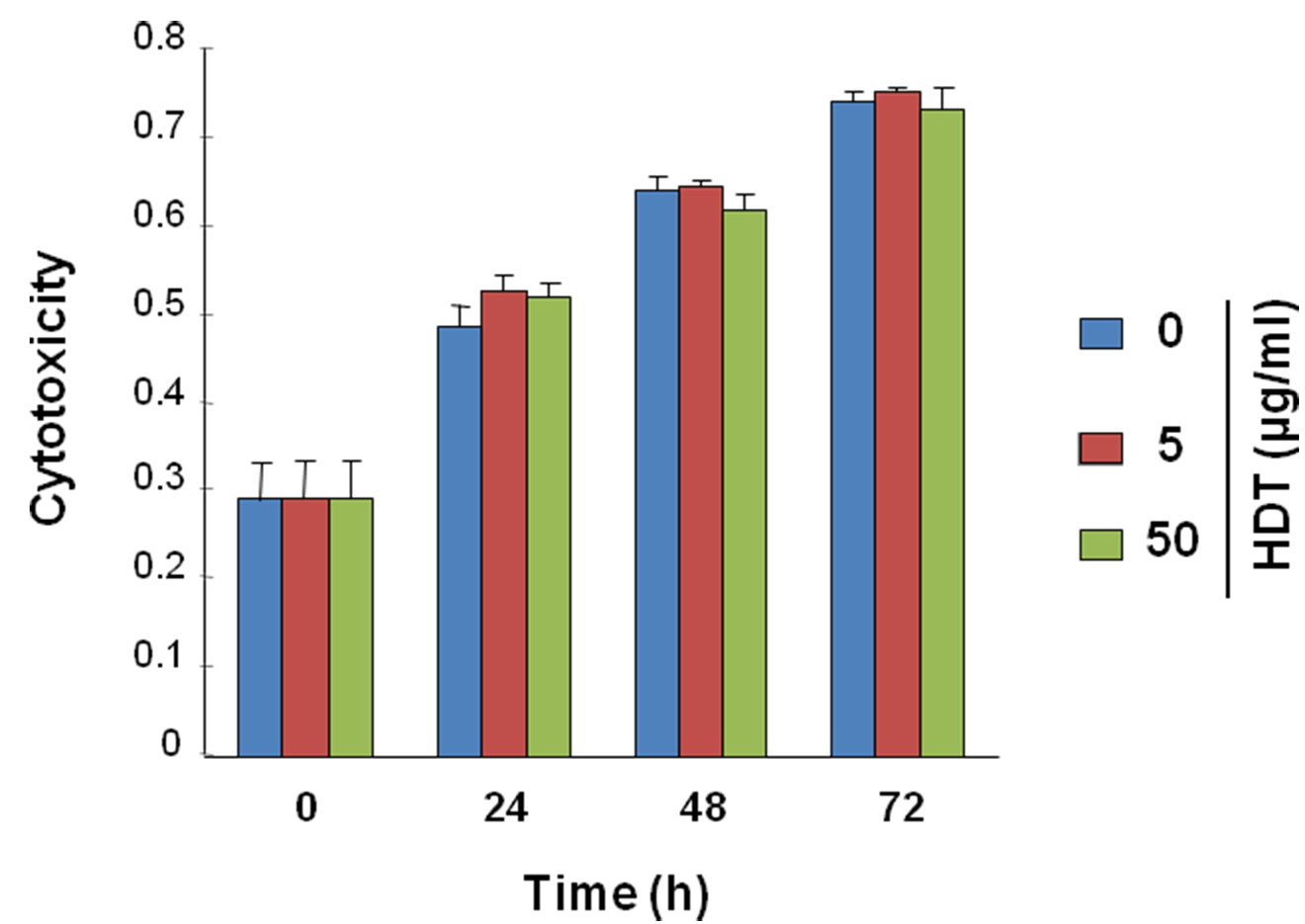

Supplement: Figure S1 — HDT extract did not affect cytotoxicity in calvarial osteoblasts. Calvarial osteoblasts were treated with HDT extract for 72 h and the cytotoxicity was assessed by MTT assay (n = 3). (TIF) [file pone.0085546.s001.tif]

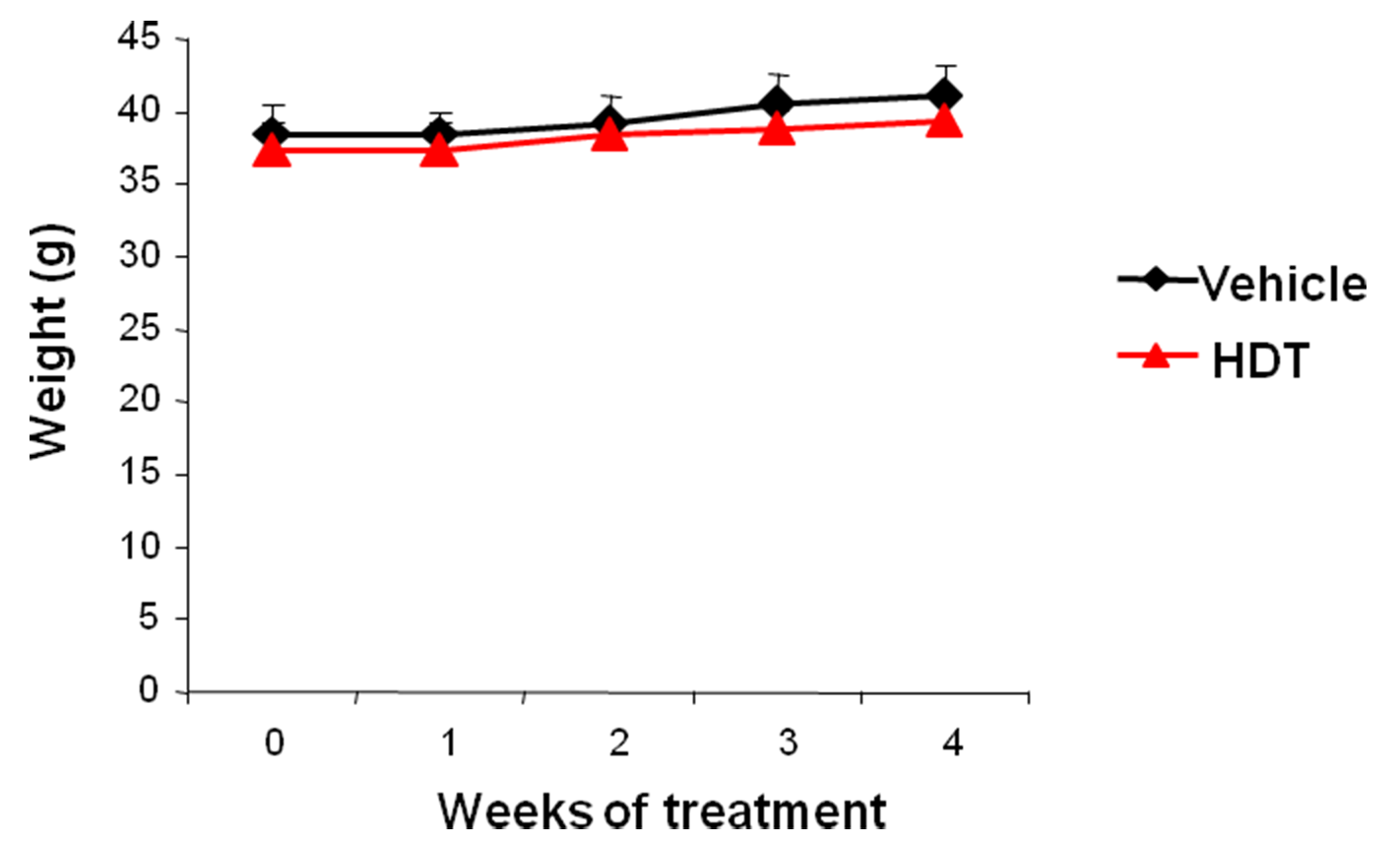

Supplement: Figure S2 — HDT extract did not induce critical changes to the weights of the mice. Weight difference of mice used in Figure 3 are presented (n = 5). (TIF) [file pone.0085546.s002.tif]

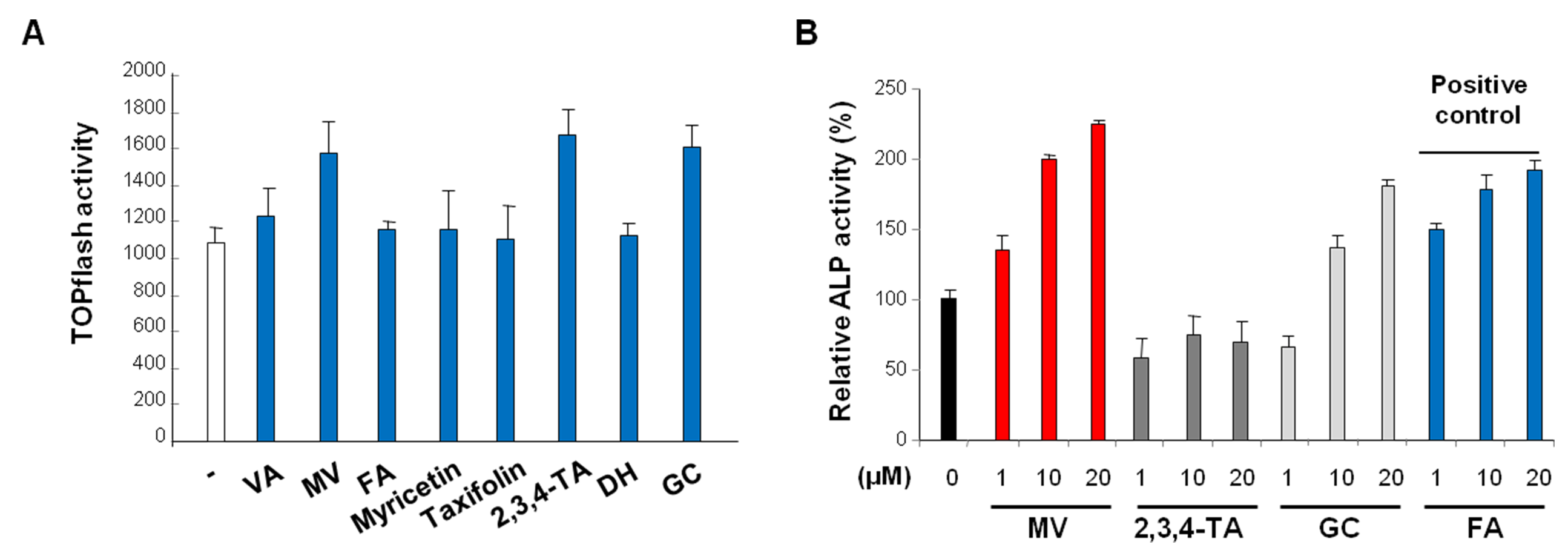

Supplement: Figure S3 — MV is an ingredient in HDT responsible for Wnt/β-catenin pathway activation and increased osteoblasts differentiation. (A) HEK293 reporter cells were treated with its 8 ingredients (20 µM) for 24 h, and subjected to TOPflash activity measurement (n = 3). (B) Calvarial osteoblasts were treated with MV, 2,3,4-TA, GC or FA for 3 days, and the cells were harvested for determination of ALP activity. ALP activity was normalized to the DMSO (control), and FA was used as a positive control (n = 3). (TIF) [file pone.0085546.s003.tif]

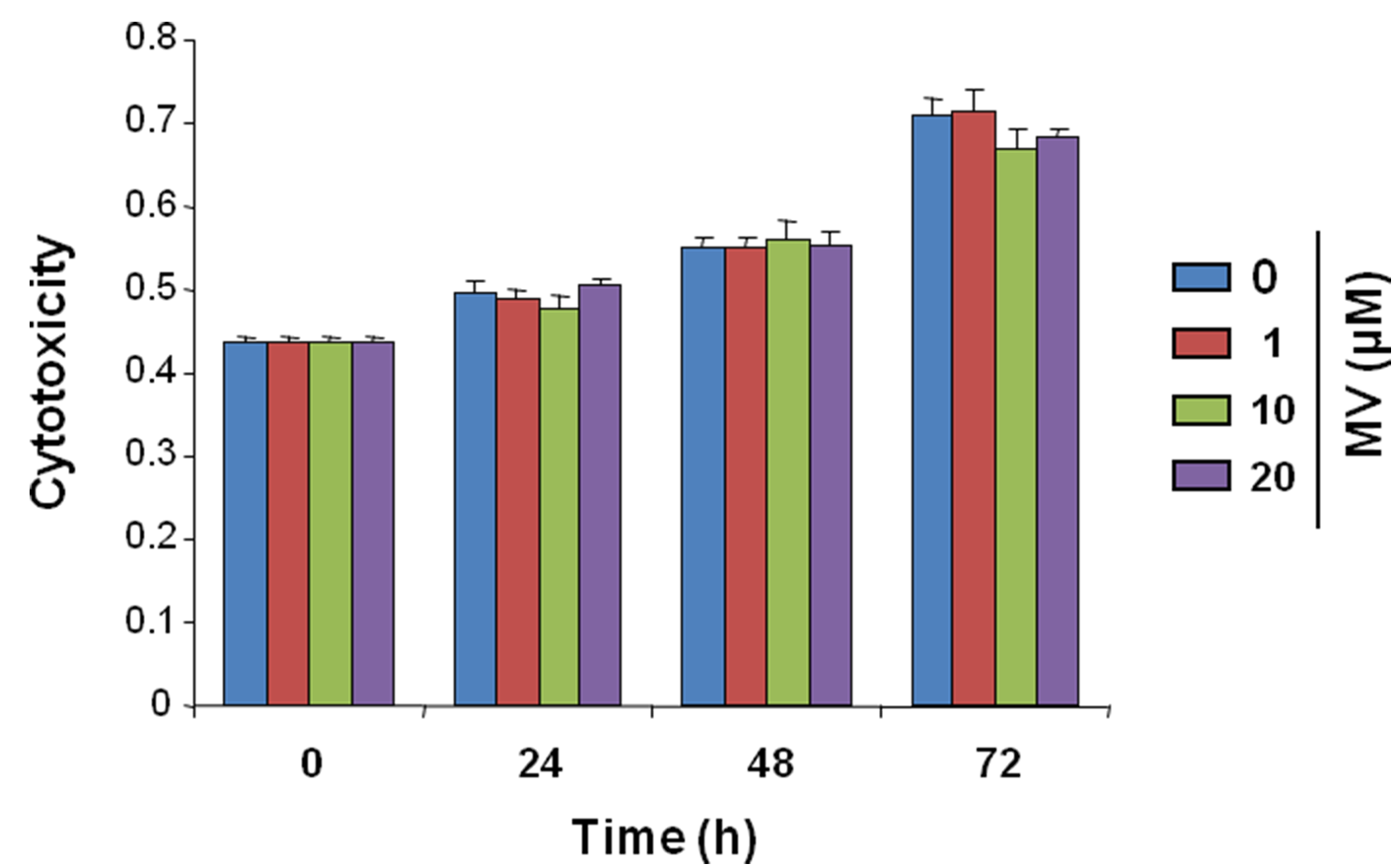

Supplement: Figure S4 — MV did not affect cytotoxicity in calvarial osteoblasts. Calvarial osteoblasts were incubated with MV for 72 h and the cytotoxicity of MV was determined by MTT assay (n = 3). (TIF) [file pone.0085546.s004.tif]

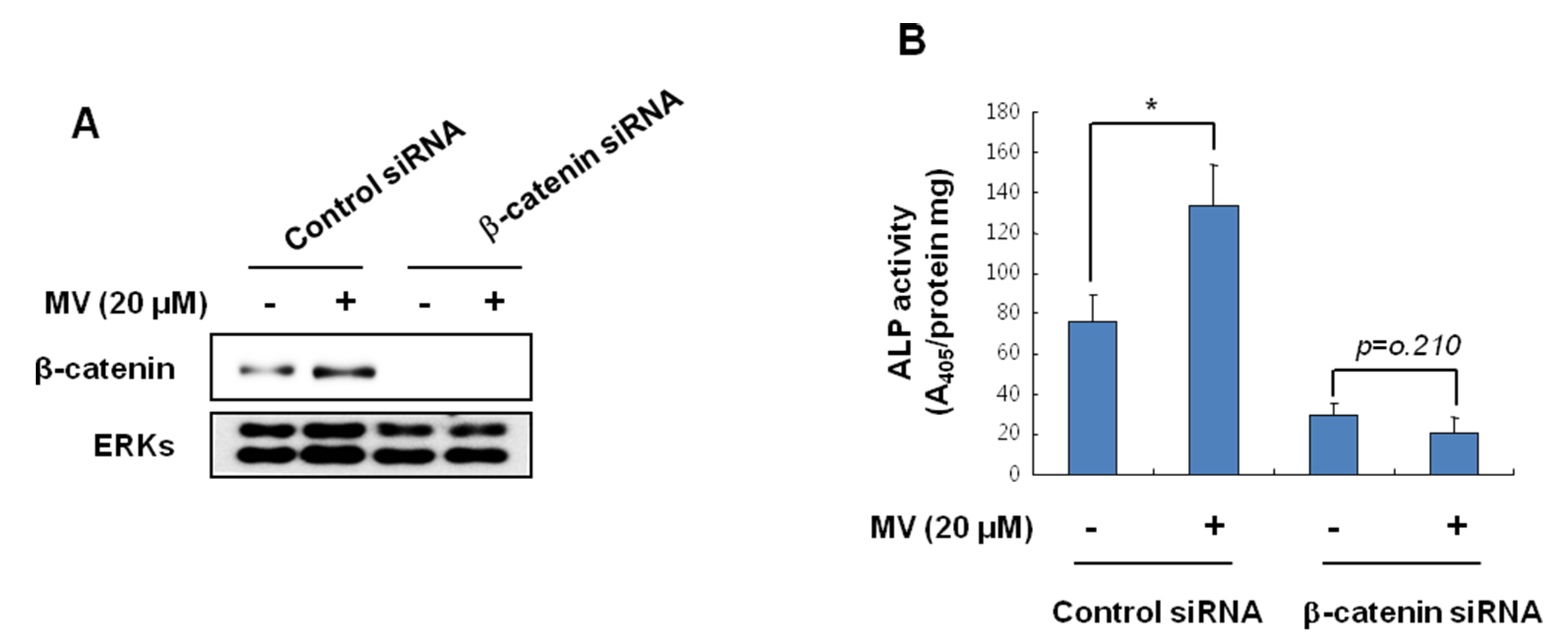

Supplement: Figure S5 — MV increases ALP activity via activation of Wnt/β-catenin pathway in MC3T3E1 cell lines. (A–B) The siRNA for β-catenin or a control (siRNA for GFP) was transfected into MC3T3E1 cells. After 12 h, MV was treated with differentiation media for 72 h and the cells were subjected to immunoblotting (A) and measurement of ALP activity (B; n = 3). (TIF) [file pone.0085546.s005.tif]

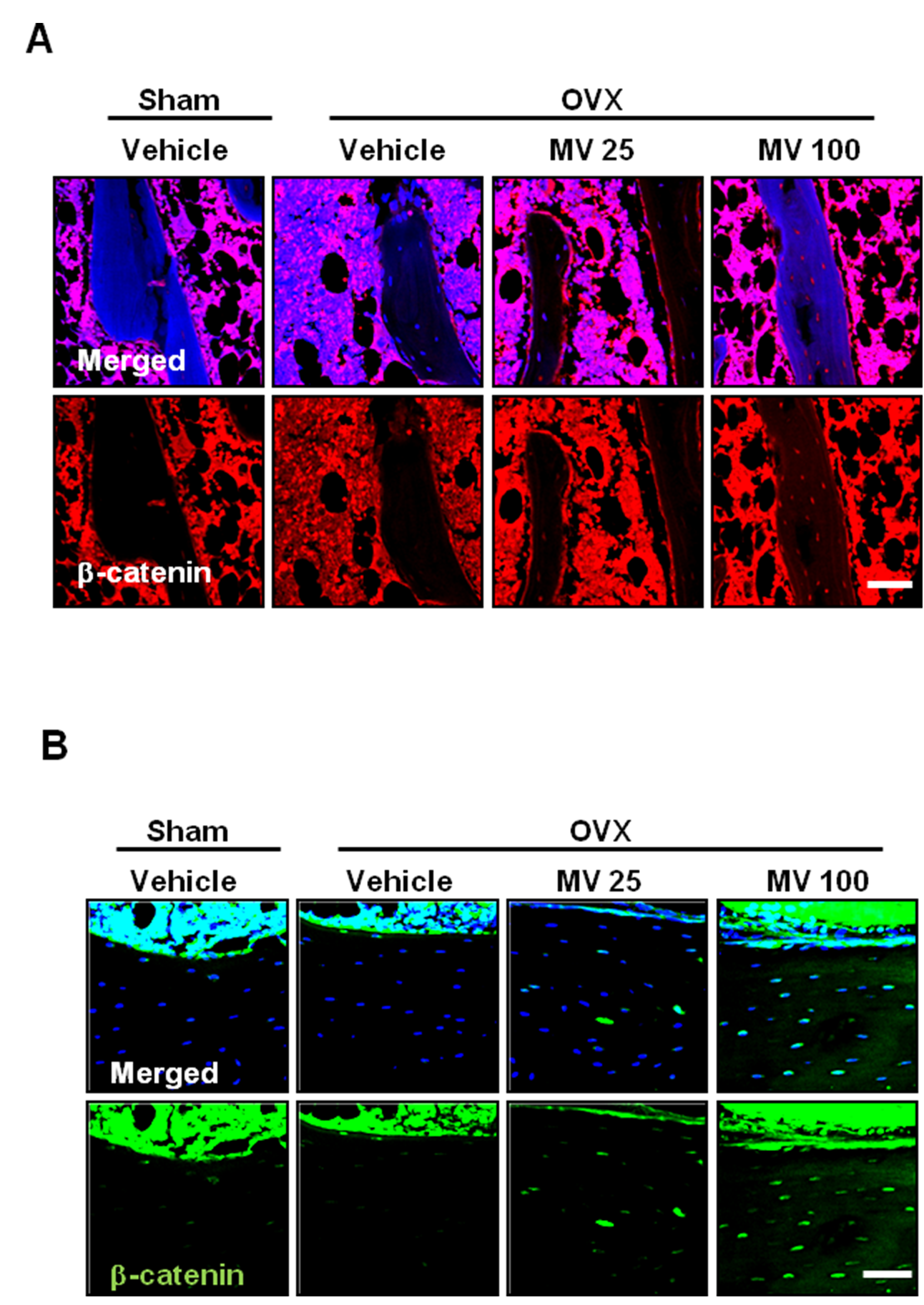

Supplement: Figure S6 — MV increases β-catenin expression at femur. (A–B) Data are shown from mice used in Fig. 5. (n = 5). Representative images of IHC staining for β-catenin in trabecular (A) and cortical (B) bone at femur. Scale bars, 50 µm. (TIF) [file pone.0085546.s006.tif]

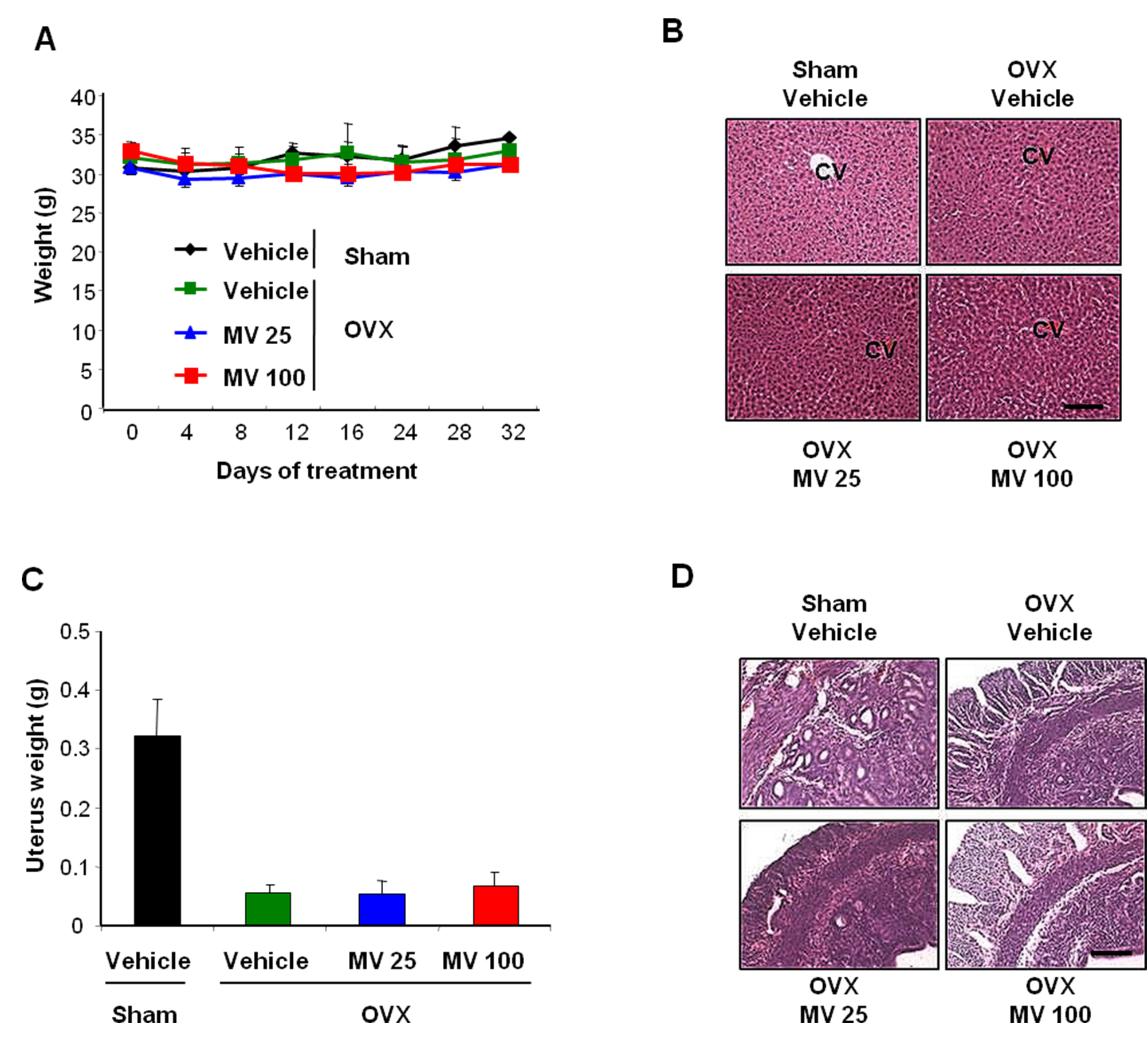

Supplement: Figure S7 — MV causes no critical abnormalities in weight, live, and uterine. (A–D) Data are shown from mice used in Fig. 5. (n = 5). (A) The weights of mice were measured every 4 days. (B) Liver tissue from the mice was stained with H&E (CV; central veins). (C) The uteri were isolated from mice, and their wet weights were measured after sacrifice. (D) The uteri in (C) were subjected to H&E staining. (B, D) Scale bars, 100 µm. (TIF) [file pone.0085546.s007.tif]
